# Evaluation of the yield of post-clipping angiography and nationwide current practice

**DOI:** 10.1007/s00701-019-03834-3

**Published:** 2019-02-19

**Authors:** N. Scheer, R. Ghaznawi, M. A. A. van Walderveen, R. W. Koot, P. W. A. Willems

**Affiliations:** 1 Department of Neurosurgery, Leiden University Medical Center, Leiden, the Netherlands; 2 Department of Radiology, Leiden University Medical Center, Leiden, the Netherlands; 3 Department of Neurosurgery, University Medical Center Utrecht, Internal Postage: G03.124, PO-box 85500, 3584 CX Utrecht, The Netherlands

**Keywords:** Saccular aneurysm, Ruptured, Unruptured, Surgery, Residual, Retreatment

## Abstract

**Background:**

Surgical treatment of intracranial saccular aneurysms aims to prevent (re)hemorrhage by complete occlusion of the aneurysmal lumen. It is unclear whether routine postoperative imaging, to assess aneurysmal occlusion, is necessary since intraoperative assessment by the neurosurgeon may be sufficient. We assessed routine clinical protocols for post-clipping imaging in the Netherlands and determined whether intraoperative assessment of aneurysm clippings sufficiently predicts aneurysm residuals.

**Methods:**

A survey was conducted to assess postoperative imaging protocols in centers performing clipping of intracranial aneurysms in the Netherlands (*n* = 9). Furthermore, a retrospective single-center cohort study was performed to determine the predictive value of intraoperative assessment of aneurysm occlusion in relation to postoperative digital subtraction angiography (DSA) findings, between 2009 and 2017.

**Results:**

No center performed intraoperative DSA in a hybrid OR, routinely. Respectively, four (44.4%), seven (77.8%), and three (33.3%) centers did not routinely perform early postoperative imaging, late follow-up imaging, or any routine imaging at all. Regarding our retrospective study, 106 patients with 132 clipped aneurysms were included. There were 23 residuals ≥ 1 mm (17.4%), of which 10 (43.5%) were unexpected. For the presence of these residuals, intraoperative assessment showed a sensitivity of 56.5%, a specificity of 86.2%, a positive predictive value of 46.4%, and a negative predictive value of 90.4%.

**Conclusions:**

There is lack of consensus regarding the post-clipping imaging strategy in the Netherlands. Since intraoperative assessment is shown to be insufficient to predict postoperative aneurysm residuals, we advocate routine postoperative imaging after aneurysm clipping unless this is not warranted on the basis of patient age, clinical condition, and/or comorbidity.

## Introduction

Subarachnoid hemorrhage (SAH) is a severe subtype of stroke. Not only does it carry significant morbimortality [21, 28], it also affects patients at a mean age of 57 years [21] leading to loss of many years of productive life. Intracranial ruptured aneurysms are the underlying cause in 85% of patients [21]. Treatment of intracranial saccular aneurysms, with or without prior SAH, primarily aims to prevent (re)bleeding by complete occlusion of the aneurysmal lumen. The current treatment of intracranial aneurysms consists of microsurgical clipping or endovascular treatment (coiling). Although endovascular treatment is performed in the majority of patients, microsurgical clipping still has some advantages and is the preferred treatment in a selective group of patients [14]. Successful clipping is defined as the complete and permanent exclusion of the entire aneurysm from the arterial circulation. The necessity for post-clipping imaging depends on the level of certainty, intraoperatively, whether complete occlusion has been achieved.

Post-clipping imaging, if necessary, is most accurate with digital subtraction angiography (DSA) [26]. It is considered the gold standard to identify residual filling of the aneurysmal lumen, which may necessitate additional follow-up and/or treatment [20]. However, performing DSA routinely adds risk [29] and burden to a majority of patients whose aneurysms prove to be adequately obliterated. A less invasive alternative is computerized tomographic angiography (CTA), but this imaging technique has been shown to be less sensitive [25]. With the number of reoperations being small [5], no consensus currently exists regarding the implementation of routine postoperative imaging, either DSA or CTA.

We hypothesized that the surgeon’s intraoperative assessment is sufficient to determine aneurysm occlusion. If so, this would render routine postoperative DSA superfluous (or intraoperative DSA in case of a hybrid operating room). To address this issue, we surveyed current routine clinical protocols for post-clipping imaging in the Netherlands. Furthermore, we performed a retrospective cohort study to determine to which extent intraoperative assessment of aneurysmal occlusion was in agreement with postoperative DSA results.

## Methods

### National survey regarding the use of imaging

To assess routine clinical protocols for post-clipping imaging in the Netherlands, a questionnaire was sent to a vascular neurosurgeon from each of the nine centers in the Netherlands performing microsurgical clipping of aneurysms, in 2017. In this survey, the neurosurgeons were asked what the general strategy at their center was (1) regarding early postoperative imaging (< 90 days post clipping) and (2) regarding late follow-up imaging (> 90 days post clipping). For both questions, they could choose from the following answers: (a) routine DSA, (b) routine CTA, (c) DSA if indicated, (d) CTA if indicated, or (e) no imaging. If the neurosurgeon chose option c or d (imaging if indicated) regarding early postoperative imaging, they were asked to specify the indication(s) for performing imaging.

In December of 2018, the same vascular neurosurgeons were asked whether their center had a hybrid operating room and, if so, whether it was routinely used to perform intraoperative DSA during or after aneurysm clipping?

### Retrospective study evaluating intraoperative assessment

#### Research design and inclusion criteria

We performed a retrospective single-center cohort study, including all patients with either a ruptured or unruptured aneurysm, who were treated microsurgically at our center, between January 2009 and August 2017, and who underwent a postoperative DSA within 90 days after surgery. Year 2009 was chosen as starting point as post-clipping DSA was not routine in our center until that time. Since then, its use leveled off at 68% of the cases, on average.

#### Data collection

Clinical information was obtained from electronic and paper medical records. The collected information included patient demographics (age, gender), risk factors for aneurysm formation (hypertension, tobacco abuse, alcohol abuse, drug abuse, positive family history of intracranial aneurysms, history of SAH), aneurysm location, aneurysm rupture, timing of aneurysm surgery (emergency or elective setting), rebleeds, timing of postoperative DSA, and clinical consequence of postoperative DSA. In patients with multiple aneurysms, each aneurysm being clipped was noted as a separate event. In cases of SAH, patient admission status was reconstructed from the records, using the Hunt and Hess (HH) scale and the World Federation of Neurosurgical Societies (WFNS) scale.

Postoperative DSA was performed with standard biplane fluoroscopy equipment (Infinix, Toshiba Medical Systems, Japan) and consisted of at least anteroposterior, lateral, and rotational images of the territory concerned, including 3D reconstructions. The DSA report of every clipped aneurysm was used to identify aneurysm residuals. The size of all residuals was subsequently measured by a neurosurgeon co-specialized in neuro-interventional radiology (PWAW).

#### Definitions of outcome measures

Operative reports were reviewed to determine whether the neurosurgeon expected a postoperative residual or not. Each report was categorized as one of the following: “not expecting a residual” (ExpN), “expecting a residual” (ExpY), or “inconclusive” (ExpI). Surgical reports mentioning complete aneurysm occlusion as well as reports with no remark about completeness of aneurysm occlusion but without any remarks regarding difficulties during surgery, were categorized as ExpN. Surgical reports specifically mentioning incomplete occlusion were categorized as ExpY. Those mentioning difficulties during surgery but without a statement on the final result were categorized as ExpI. To avoid underestimation of the number of unexpected residuals, we dichotomized between ExpN on the one hand and either ExpY or ExpI on the other in the final analysis.

Residual aneurysms at DSA were categorized as follows: no residual (*R* = 0), residual < 1 mm (*R* < 1) and residual ≥ 1 mm (*R* ≥ 1). In the final analysis, these categories were dichotomized as *R* ≥ 1 on the one hand and either *R* < 1 or *R* = 0 on the other, because residuals ≥ 1 mm have a higher risk of regrowth [6] and are, therefore, clinically relevant.

#### Statistical analysis

All analyses were performed using Statistical Package for the Social Sciences (SPSS) version 20. Normally distributed variables were expressed as mean with standard deviation (SD) and/or range, when appropriate. Skewed distributed variables were expressed as median with interquartile range (IQR). Statistical significance was defined as *P* < 0.05.

Sensitivity, specificity, positive, and negative predictive values with their 95% confidence intervals (CI) were determined for the intraoperative assessment. DSA results acted as gold standard.

Imaging is most likely to be omitted in cases when a residual is not expected (ExpN). Therefore, we decided to perform an additional analysis in this subgroup to determine whether other variables could predict the need for DSA imaging. These variables included aneurysm location, timing of aneurysm surgery, HH scale and WFNS scale. For this analysis, the variable aneurysm location was dichotomized as “aneurysm at anterior communicating or ophthalmic artery region” versus “aneurysm at other location,” since deep midline aneurysms and aneurysms at an ophthalmic artery region have an increased risk of imperfect clip placement [23].

## Results

### National survey regarding intraoperative and postoperative imaging

The response rate of the survey was 100% (9/9).

Regarding a hybrid operating room, only five centers (55.6%) had one at the beginning of 2019 and at least two of these had only recently become available. Three other centers were in the process of building one. None of the centers used the hybrid room routinely for aneurysm clipping, and only one center used it routinely for unruptured aneurysms.

**Fig. 1:**
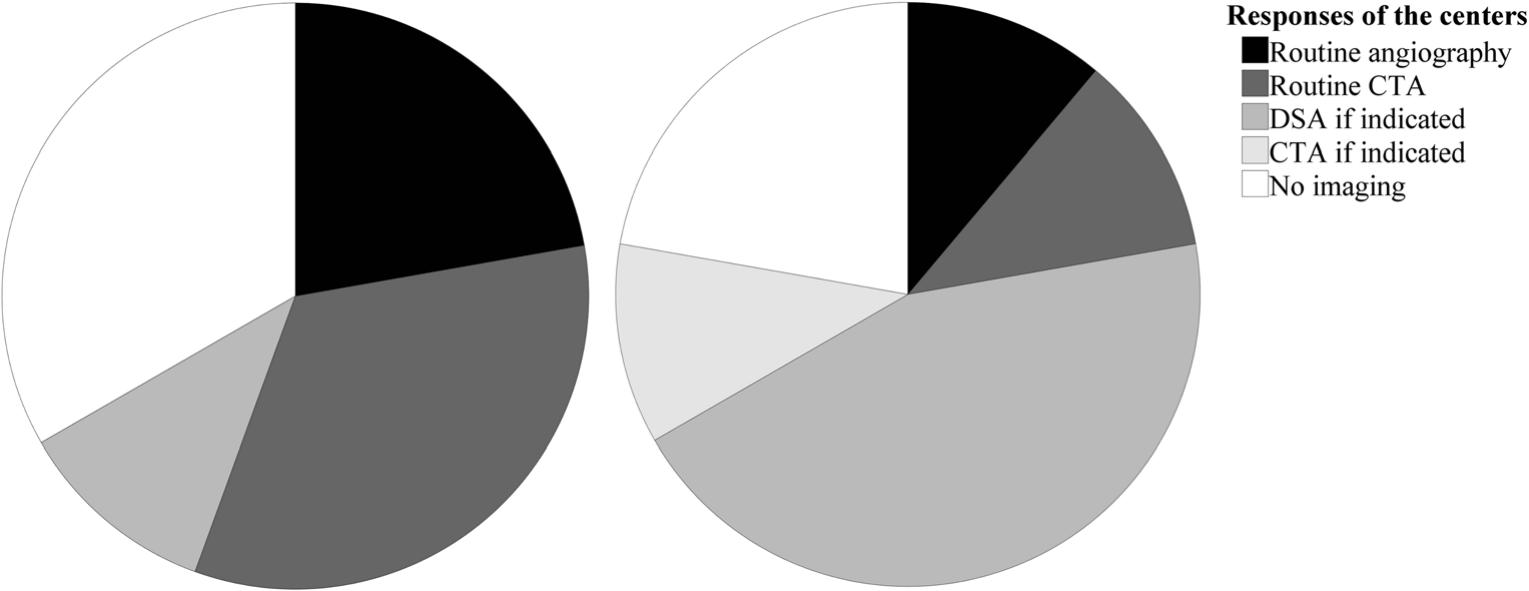
Pie charts of the responses on the national survey regarding postoperative imaging (*n* = 9). Left: early postoperative imaging (< 90 days post clipping). Right: late follow-up imaging (> 90 days post clipping). CTA computerized tomographic angiography; DSA (or 'angiography') digital subtraction angiography

The responses regarding postoperative imaging are shown in Fig. 1. One neurosurgeon answered “DSA if indicated” on the question regarding early postoperative imaging. The neurosurgeon explained that performing DSA in his center depended on the intra-operative impression of the neurosurgeon regarding a possible residual. Four centers did not routinely perform early postoperative imaging (44.4%) and seven did not routinely perform late imaging (77.8%). Three centers performed neither routine early nor routine late postoperative imaging (33.3%).

### Retrospective study evaluating intraoperative assessment

#### Patient population

We identified 160 patients with 198 ruptured and unruptured aneurysms, clipped between January 2009 and August 2017. Thirty-seven patients (23.1%) with 40 aneurysms (20.2%) were excluded, due to lack of a postoperative DSA. Reasons for not performing an angiography were as follows: poor clinical condition (20 patients), relocation to primary institution prior to DSA (5 patients), DSA deemed too risky (1 patient), renal failure (1 patient), DSA refused by the patient (1 patient), or an unknown reason (9 patients). This resulted in 123 patients, with 158 aneurysms. Of these patients, 106 cases (86.2%) with 132 aneurysms (83.5%) underwent angiography within 90 days post clipping. These patients were included in the study. The baseline characteristics of the patients are shown in Table 1. The characteristics of the 132 clipped aneurysms are presented in Table 2. The median time to DSA was 5.0 days after surgery (IQR 2.3–9.0 days). The majority of patients (68.9% of all aneurysms) had a postoperative DSA within 1 week after clipping.

**Table 1:** Patient characteristics (*n* = 106 patients)

Characteristic	Value (%)
Age (years)	
Mean ± SD	55.3 ± 13.2
Range	12–79
Gender male	26 (24.5)
Presenting with SAH	69 (65.1)
WFNS grade at arrival in hospital	1: 39 (56.5)
	2: 8 (11.6)
	3: 3 (4.3)
	4: 8 (11.6)
	5: 11 (15.9)
HH grade at arrival in hospital	1: 15 (21.7)
	2: 27 (39.1)
	3: 11 (15.9)
	4: 10 (14.5)
	5: 6 (8.7)
Number of clipped aneurysms per patient	
One aneurysm	84 (79.2)
Two aneurysms	18 (17.0)
Three aneurysms	4 (3.8)
Risk factors aneurysm formation	
Hypertension	40 (37.7)
Tobacco abuse	41 (38.7)
Alcohol abuse	12 (11.3)
Drug abuse	4 (3.8)
Positive family history of intracranial aneurysms	6 (5.7)
History of SAH	3 (2.8)

*SAH*, subarachnoid hemorrhage; *WFNS*, World Federation of Neurosurgical Societies; *HH*, Hunt and Hess

**Table 2:** Aneurysm characteristics (*n* = 132 aneurysms)

Characteristic	Value (%)
Location	
ACA (excluding ACoA)	9 (6.8)
AChA	8 (6.1)
ACoA	20 (15.2)
ICA	6 (4.5)
MCA	67 (50.8)
PCoA	18 (13.6)
PICA	3 (2.3)
VA	1 (0.8)
Ruptured	66 (50.0)
Unruptured	66 (50.0)
Of which clipped in emergency setting	23 (34.8)
Rebleed after clipping	0

*ACA*, anterior cerebral artery; *AChA*, anterior choroidal artery; *ACoA*, anterior communicating artery; *ICA*, internal carotid artery; *MCA*, middle cerebral artery; *PCoA*, posterior communicating artery; *PICA*, posterior inferior cerebellar artery; *VA*, vertebral artery

#### Residuals

Evaluation of DSA imaging of the 132 clipped aneurysms yielded 23 aneurysms labeled *R* ≥ 1 (17.4%), 11 aneurysms with *R* < 1 (8.3%), and 98 aneurysms with *R* = 0 (74.2%). Characteristics of the *R* ≥ 1 aneurysms are shown in Table 3.

**Table 3:** Characteristics of residuals ≥ 1 mm (*n* = 23)

Characteristic	Value (%)
Location	
AChA	2 (8.7)
ACoA	7 (30.4)
MCA	9 (39.1)
PCoA	5 (21.7)
Size	
Median (IQR)	1.5 (1.2–2.5)
Range	1.0–14.0
Ruptured aneurysm	17 (73.9)
Unruptured aneurysm	6 (26.1)
Of which clipped in emergency setting	2 (33.3)

*AChA*, anterior choroidal artery; *ACoA*, anterior communicating artery; *MCA*, middle cerebral artery; *PCoA*, posterior communicating artery; *IQR*, interquartile range

#### Evaluation of intraoperative assessment and postoperative angiography

Of the 23 residuals ≥ 1 mm in size, 10 were expected (43.5%) based on the intraoperative assessment, 10 were unexpected (43.5%), and in 3 the assessment was inconclusive (13.0%). The results of the intraoperative assessments and postoperative angiographies are shown in Table 4.

**Table 4:** Results of the operative reports and postoperative DSA (*n* = 132)

		Postoperative DSA		Total
		*R* ≥ 1	*R* = 0 or *R* < 1	
Operative report	ExpY or ExpI	13	15	28
	ExpN	10	94	104
	Total	23	109	132

*DSA*, digital subtraction angiography; *ExpI*, an operative report categorized as “inconclusive;” *ExpN*, is an operative report categorized as “not expecting a residual;” *ExpY*, an operative report categorized as “expecting a residual;” *R* = 0, no residual, *R* < 1, residual < 1 mm, *R* ≥ 1, residual ≥ 1 mm

The sensitivity of the intraoperative assessment for the presence of a residual ≥ 1 mm was 56.5% (95% CI = 0.35–0.76), its specificity 86.2% (95% CI = 0.78–0.92), its positive predictive value 46.4% (95% CI = 0.28–0.66), and its negative predictive value 90.4% (95% CI = 0.83–0.95).

#### Clinical consequences of the residuals

The clinical consequences of finding a residual on postoperative DSA were as follows: follow-up DSA in 18 aneurysms (78.3%), endovascular re-treatment (coiling) in 1 aneurysm (4.3%), surgical re-treatment in 1 aneurysm (4.3%), and no consequence in 3 aneurysms (13.0%).

When these consequences were dichotomized on the basis of whether the residual had been expected, no obvious differences were perceived. Out of 10 residuals that were labeled ExpN, 8 had a follow-up DSA (80%), and one was retreated endovascularly (10%), while out of 13 residuals that were labeled either ExpY or ExpI, 10 had a follow-up DSA (77%), and one was retreated surgically (8%).

#### Subgroup analysis

In the ExpN subgroup, logistic regression analysis did not reveal significant associations between timing of aneurysm surgery (*P* = 0.06), location (*P* = 0.41), HH scale (*P* = 0.21) or WFNS scale (*P* = 0.52) and *R* ≥ 1. In other words, the occurrence of an aneurysm residual of 1 mm or larger that was not to be expected from the intraoperative assessment was not associated with any of these parameters.

#### Case presentations

We present details of the two cases with residuals requiring re-treatment.

**Fig. 2:**
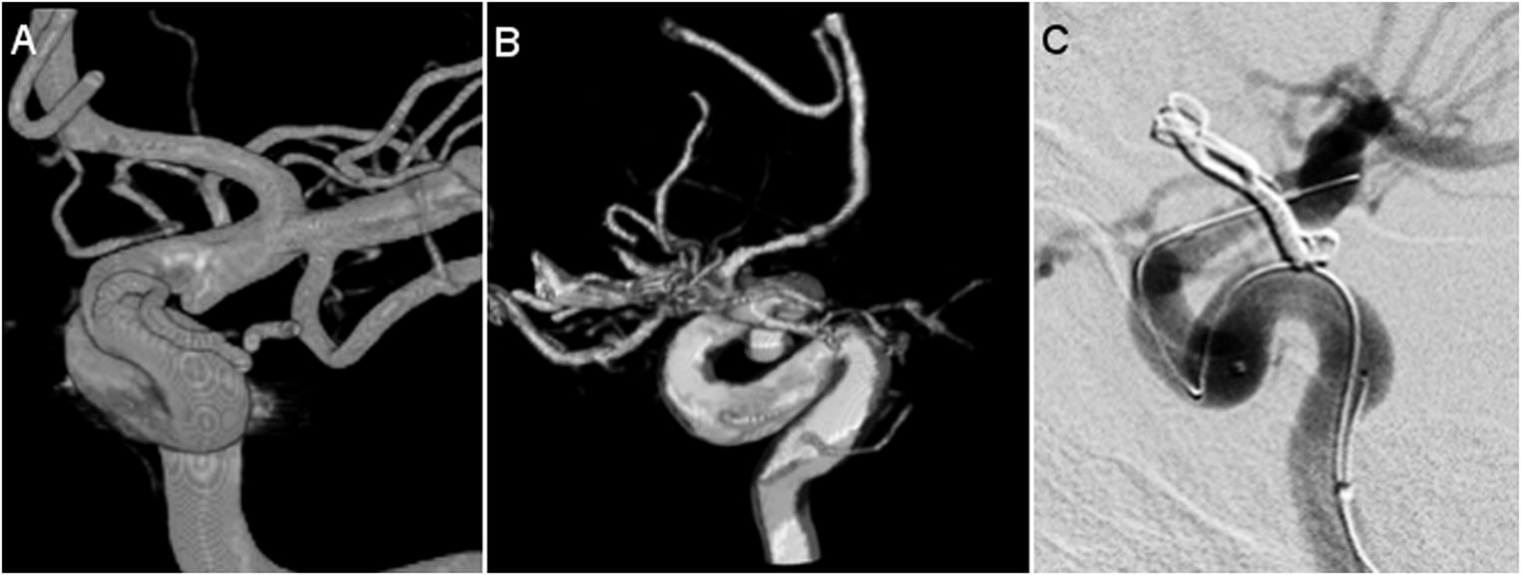
DSA images of a patient with a postoperative residual requiring coiling. **a** Preoperative 3D-reconstruction of the rotational angiogram demonstrating a ruptured, 3.7 mm, left PCoA aneurysm. **b** Postoperative 3D-reconstruction of the rotational angiogram showing residual filling. **c** DSA (lateral projection) showing the result of coiling. DSA digital subtraction angiography, PCoA posterior communicating artery

**Fig. 3:**
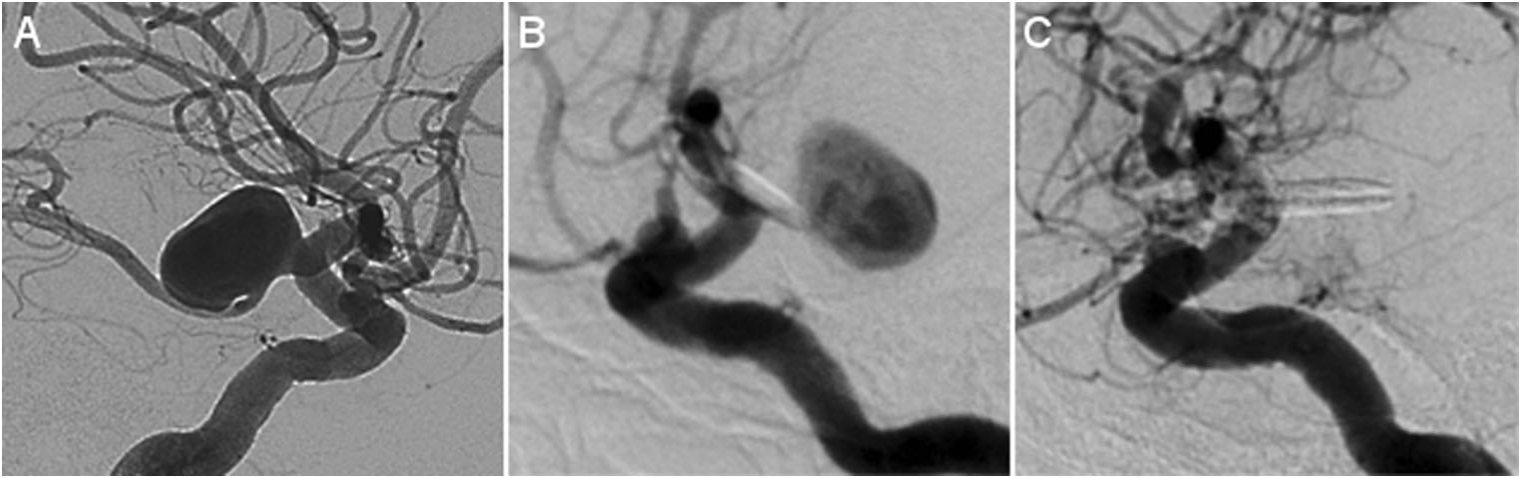
DSA images of a patient with a postoperative residual requiring re-clipping. **a** DSA (lateral projection) demonstrating a ruptured, 14.0 mm, PCoA aneurysm. **b** Postoperative DSA (lateral projection) showing residual filling of the entire aneurysm. **c** DSA (lateral projection) showing successful re-clipping. DSA digital subtraction angiography, PCoA posterior communicating artery

- Case 1. A 54-year-old woman presented with SAH, WFNS grade 1. DSA revealed a 3.7 mm, broad-based, posterior communicating artery (PCoA) aneurysm. The aneurysm was clipped and, based on the operative report, the neurosurgeon did not expect a residual. After the operation, the patient suffered from bradyphrenia due to vasospasm, for which induced hypertension was initiated on the intensive care unit. Postoperative DSA was performed 5 days later, showing residual filling of the aneurysm and severe angiographic vasospasm. The residual aneurysm was 2.3 mm in size (*R* ≥ 1). Within 2 days, the aneurysm was successfully coiled. DSA images of this patient are shown in Fig. 2.
- Case 2. A 67-year-old woman arrived in the hospital with SAH, WFNS grade 1. CTA revealed a lobulated, 14.0 mm, PCoA aneurysm in the context of a fetal origin of the posterior cerebral artery. The aneurysm was clipped. In his operative report, the neurosurgeon reported multiple problems during surgery and finally described “almost complete occlusion” of the aneurysm. Surprisingly, postoperative DSA demonstrated complete, albeit slowed, filling of the entire aneurysm through a small remaining channel at the level of the aneurysm neck. Successful re-clipping followed the next day. Key images are shown in Fig. 3.

## Discussion

We aimed to assess routine clinical protocols regarding imaging after intracranial aneurysm clipping in the Netherlands and to determine the predictive value of the intraoperative assessment for a postoperative residual. In a national survey, clinical protocols varied widely. In a single-center retrospective evaluation, the negative predictive value of intraoperative assessment was shown to be only 90.4%.

### Routine clinical protocols for post clipping imaging

Almost half of the Dutch centers treating intracranial aneurysms (4/9) did not routinely perform early postoperative imaging and a third (3/9) performed neither routine early nor routine late imaging. Apparently, these centers expected incomplete aneurysmal occlusion to be evident during surgery, in contrast to our current findings. Of the five centers performing routine early postoperative imaging, three used CTA and two used DSA. This represents the debate in literature regarding the optimal type of imaging for clipped aneurysms. Multiple studies suggest that postoperative assessment of clipped aneurysms can be performed with CTA, as an alternative for DSA [7, 8, 12], or suggest to limit the use of DSA to certain indications [10, 17, 27]. However, a recent meta-analysis, with a total of 487 aneurysms, showed that CTA had a pooled sensitivity of only 71% and specificity of 94% for identifying residual or recurrent aneurysms in comparison to DSA [25]. For aneurysm residuals < 2 mm, the sensitivity has been shown to be even lower [26]. Although residuals < 1 mm have been shown to have a lower regrowth risk [6], all residuals ≥ 1 mm should be considered clinically relevant for risk of regrowth and hemorrhage [6, 13]. Taking this into account, we believe an optimal imaging strategy should include at least one DSA.

When a hybrid OR is available, immediate intraoperative post clipping DSA could substitute for postoperative imaging, but our results clearly indicate that this is not yet routine practice in our country.

### Predictive value of intraoperative assessment

The residual rate after clipping, either expected or unexpected, was 17.4% in our series. This is slightly higher compared to previous studies (Table 5). With regard to the operative reports, which we regard to be an indicator of the surgeons intraoperative impression, the predictive value for a postoperative residual was very limited. The negative predictive value of the intraoperative assessment for a clinically relevant residual was only 90.4%. This implies that of all the cases where the surgeon would likely find postoperative imaging unnecessary, almost 10% would still contain a residual. Apparently, it is not straightforward for a surgeon to assess complete aneurysm occlusion. This is in accordance with previous studies [16, 19, 20]. However, our results are relevant since the study from MacDonald et al. [19] dates from before the use of rotational angiography, Kivisaari et al. [16] did not clarify how the distinction ‘unexpected’ was made and Meyer et al. [20] found a much lower incidence of unexpected residuals. Moreover, the fact that one of our unexpected residuals required early re-treatment, also suggests that routine postoperative imaging is important.

**Table 5:** Publications of postoperative residuals on digital subtraction angiography

Authors and year	Aneurysms	% Residuals
Acevedo et al. (1997) [1]	267	6.3
Akyüz et al. (2002) [2]	186	7.0
Akyüz et al. (2004) [3]	166	4.2
Bernat et al. (2017) [4]	37	24.3*
Brown et al. (2017) [5]	758	7.8
Burkhardt et al. (2017) [6]	346	4.6
David et al. (1999) [9]	147	8.2
Dellaretti et al. (2017) [11]	105	13.3
Feuerberg et al. (1987) [13]	715	3.9
Hollin et al. (1973) [15]	55	5.5
Kivisaari et al. (2004) [16]	808	12.0
Le Roux et al. (1998) [18]	637	5.7
Macdonald et al. (1993) [19]	78	10.3
Meyer et al. (2004) [20]	384	4.9
Proust et al. (1997) [22]	44	2.3
Rauzzino et al. (1998) [23]	312	4.2
Sindou et al. (1998) [24]	305	5.9

*Of which 13.5% were considered a neck remnant

### Study limitations

With regard to the survey, we asked one neurosurgeon from each center to describe the general strategy at that center. This may lead to a slight misrepresentation as surgeons within one center may also use different imaging strategies. However, this would only further underline the lack of consensus and need for an evidence based guideline.

Limitations of our evaluation of the intraoperative assessment include its retrospective nature. We cannot exclude the possibility that we have overestimated the number of unexpected residuals. It is possible that the neurosurgeon anticipated a residual but did not document this clearly in the operative report. We attempted to minimize this risk by grouping inconclusive reports with the ones that expected a residual. Furthermore, our study lacks long-term follow-up to demonstrate the natural history of small residuals and their clinical consequences.

### Significance of follow-up DSA imaging

The main goal of postoperative angiography is to eliminate the risk of a rebleed from a residual aneurysm by allowing follow-up and/or retreatment. Therefore, to decide whether postoperative DSA should be performed routinely, one should balance the risk of a hemorrhage from a (growing) residual with the inherent risk of follow-up DSA and subsequent treatment. The data for this analysis cannot be derived solely from our current study. For an aneurysm residual, the annual risk of regrowth is 2.1% per year [6] and the annual risk of hemorrhage lies around 0.8% [13] and 1.5% [9]. Assuming that the risk of hemorrhage is 1.0% and is constant throughout the remainder of one’s life, the cumulative risk of rupture for a 55 year-old, the average age in our series, with a life expectancy of 83 years is 25% (1–0.99^28^). If we take into account that there is only a 9.6% chance of an unexpected residual, the rate found in our series, the a priori cumulative lifetime risk of rupture should be estimated at 2.4% over a period of 28 years. On the other hand, the risk of permanent neurological sequelae from DSA for this patient population is estimated at only 0.13% [29]. Based on these numbers, we would advocate follow-up DSA imaging unless age, clinical condition, and/or comorbidity sway the balance into another direction or if the residual rate at a specific institution is known to be significantly lower. The latter would require knowledge of ones own residual rate, based on routine DSA analysis. A more detailed and in-depth analysis would require more data on natural history and clinical consequences of residuals and should be part of future research efforts.

## Conclusions

There is lack of consensus regarding the postoperative imaging strategy after intracranial aneurysm clipping in centers throughout the Netherlands. Intra-operative assessment was shown to be insufficient to predict postoperative aneurysm residuals while post-clipping DSA had a substantial yield, at our center. Thus, we believe postoperative imaging should be routine after aneurysm clipping and we suggest that this includes at least one DSA, unless this is not warranted on the basis of patient age, clinical condition and/or comorbidity, or if a center has a documented significantly lower residual rate.
